# Clinicopathological study of patients with head and neck sarcomas

**DOI:** 10.1590/S1808-86942011000300019

**Published:** 2015-10-19

**Authors:** Isabela Alves Pacheco, Ana Paula Negreiros Nunes Alves, Mário Rogério Lima Mota, Paulo César de Almeida, Marcelo Esmeraldo Holanda, Eric Fernandes de Souza, Fabricio Bitu Sousa

**Affiliations:** 1 MSc in Dentistry, DDS -Instituto de Previdência do Município de Fortaleza; 2 PhD in Pharmacology, Adjunct Professor of oral pathology - Pharmacy, Dentistry and Nursing School - Federal University of Ceará; 3 PhD in Pharmacology, Adjunct Professor of Oral Pathology and Stomatology - Pharmacy, Dentistry and Nursing School - Federal University of Ceará; 4 PhD in Public Health; Adjunct Professor of the Health Sciences Center of the State University of Ceará; 5 Expert in Oncologic Surgery, with emphasis in Head and Neck Surgery, Assistant Physician at the Head and Neck Clinic at the Santa Casa de Misericórdia Hospital; 6 Expert in Head and Neck Surgery - Cancer Institute of Ceará; 7 PhD in oral pathology, Adjunct Professor of Oral Pathology and Stomatology - Pharmacy, Dentistry and Nursing School - Federal University of Ceará; Universidade Federal do Ceará

**Keywords:** drug therapy, head and neck neoplasms, radiotherapy, sarcoma

## Abstract

Sarcomas are rare tumors, mainly stemming from the embryonic mesoderm, with a high grade of morbidity and mortality.

**Objective:**

To carry out a retrospective study of head and neck sarcoma cases between 1999 and 2008 in three specialized centers in the city of Fortaleza.

**Materials and Methods:**

Data collection was based on the charts of the patients in the study. For statistical analysis purposes we used the chi-square associations and the z test for proportions.

**Results:**

We found records of 36 patients, and the most affected ones were adult brown males, in the age range between 20 and 59 years - mean age of 39.7. The man/woman ratio was 1.76:1. The most prevalent histological type was the rhabdomyosarcoma and their most common locations were the face and the neck. Most of the sample was made up of live patients without evidence of the disease in the last visit - 41.6% of the cases. The most common treatment modes were the combination of surgery + radiotherapy + chemotherapy and surgery + radiotherapy, with 27.8% of the cases each.

**Conclusion:**

Sarcomas have a great histological variability and may have numerous locations. Since these are rare and not well-known lesions, new epidemiological studies must be carried out in order to enhance our understanding of the disease.

## INTRODUCTION

Sarcomas are a very heterogeneous group of rare tumors which stem mainly from the embryonic mesoderma^1^, they may be characterized as tumors stemming mainly from bones, cartilage or soft tissue^2^, such as fibrous, fat, muscular, synovial, vascular or neural tissue^3^, ^4^. They have an incidence of 7,400 new cases and 4,200 deaths per year in the US alone^5^, ^6^, being an important group of tumors because of their high morbidity and mortality rates.

Sarcomas represent only 1% of all cancer cases^1^, ^7^. Less than 20% of all sarcomas affect the head and neck sites in adults^3^ and 35% affect pediatric patients. The man/woman ratio is 1.42:1^2^.

Oral lesions are even rarer. Gorsky and Epstein,^3^ in a study on sarcomas and soft tissues, noticed that oral sarcomas represented 0.14% of all head and neck cancers, which shows how rare these tumors are. Lajer et al.^8^ reported from their study that oral sarcomas represented 27.7% of these head and neck tumors, and Penel et al.^9^ reported a 10.7% frequency.

Sarcomas vary considerably in location and histopathological presentation^1^, ^3^, ^9^. More than 50 histology subtypes have been identified^1^, ^2^. They have a large spectrum of clinical activities, varying from a relatively slow growth, to an aggressive local and regional growth, with a potential do develop systemic metastases^10^.

Most head and neck sarcomas involve soft tissue, and the hard tissue sarcomas are very rare^11^, representing only 20% of the cases^7^. The most common histological types in the head and neck are the rhabdomyosarcomas, followed by malignant histiosarcomas, fibrosarcomas and neurofibrosarcomas^3^.

Head and neck sarcoma treatment is based on tumor type, stage, location, size and also patient age. Chemotherapy and radiotherapy are the usual combined treatment used^7^, ^12^, ^13^, ^14^.

Compared to other locations, head and neck sarcomas have a worse prognosis, since the entire tumor removal is impaired by its proximity to vital structures^4^, ^15^, ^16^, which increases the risk of recurrences and functional and cosmetic deformities^5^.

Thanks to its large diversity and rarity, we still know very little about sarcomas. Although rare, head and neck sarcomas cause high morbidity and mortality, and they should be known to all health care professionals, in order to have early diagnoses and more efficient treatment. This paper aims at contributing to a better understanding of this disease, establishing the clinical-pathological profile of patients with head and neck sarcomas seen in three specialized centers.

## MATERIALS AND METHODS

This is a cross-sectional, descriptive study, with retrospective data. We included patients with primary sarcomas of the head and neck, seen between 1999 and 2008 in the Head and Neck Surgery Centers of the following hospitals: Santa Casa de Misericórdia de Fortaleza, Centro Regional Integrado de Oncologia (CRIO) and Hospital do Câncer do Ceará. We took off the study those patients with Kaposi sarcomas for their clinical differences in relation to the other sarcomas, since they are associated with type 8 human virus herpes, and also patients with incomplete charts.

Patient data were collected from the records of head and neck surgeriesand the charts from the assessed centers. We studied the following variables: age, gender, race, origin, primary anatomical location of the neoplasm, pathology diagnosis, type of cancer treatment employed and patient status during the most recent medical visit. This study was submitted to the Ethics in Research Committee of the Hospital do Câncer/Instituto do Câncer do Ceará, and it was approved under protocol # 015/09.

The results were plotted in Tables and submitted to statistical analysis. We used the chi-square association test and the z test for proportions, and a *p* value lower than 0.05 was considered statistically significant.

## RESULTS

During the study period, we found 51 patients with head and neck sarcomas; ten of them were taken out because they had Kaposi sarcoma, and five because they had incomplete charts; thus, the final sample had 36 patients. The mean follow up of these patients was of 27.6 months (a 1-84 month variation).

Of this sample, 23 (63.9%) were males, and 13 (36.1%) were females, with a statistically significant difference (*p*=0.046). The man/woman ratio was 1.76:1. As far as age is concerned, the most affected age range was between 20 and 59 years, representing 41.6% of the cases, as depicted on Table 1; nonetheless, there was no statistically significant difference between the age ranges. The mean age of the patients was 39.7 (±25.1).

**Table 1:** Sample characteristics

Variable	N	%
Age range		p=0,756
0-19	11 30,6	
20-59	15	41,6
≥ 60	10	27,8
Gender		*p* = 0,046
Female	13	36,1
Male	23	63,9
Ethnicity		*p* = 0,004
White	9	27,7
Brown	24	72,3
Origin		*p* = 0,999
Capital	18	50
Countryside	17	47,2
Unspecific	1	2,8

In three (8.3% of the cases), there was no information on the patient's ethnicity. Of the specified total, the most affected skin color was brown, with 72.3% of the cases, followed by whites, with 27.7%, and the difference was statistically significant (*p*=0.004). There were similarities associated with patient origin, half of them coming from the capital city and the other half coming from the country side, and we did not have information about the origin of one patient (Table 1).

As far as pathology is concerned, we found 12 histopathological variations of sarcomas, and the most common type was the rhabdomyosarcomas, representing 25% of the cases, followed by the dermatofibrosarcoma protuberans and fibrosarcoma, each one with a frequency of 13.8%. These diagnoses were settled after histopathology and immunohistochemical analyses in the research centers. The most frequent locations were the face and the neck, each representing 22.2% of the sarcomas, followed by the scalp, with 19.4% of the cases. Intraoral lesions had an incidence frequency of 13.8%. Table 2 shows the sample distribution as to histological type and tumor location.

**Table 2:** Sample distribution according to histology type and location.

Histology type	Location							Total (%)
	Face	Oral Cavity	Scalp	Orbit	Neck	Gnathic bones	Parapharyngeal space	
Rhabdomyosarcomas	4	1	-	1	3	-	-	9 (25)
Dermatofibrosarcoma	1	-	3	-	1	-	-	5 (13,8)
Fibrosarcoma	-	2	3	-	-	-	-	5 (13,8)
Malignant Fibro-histiocytoma	2	-	-	-	1	-	-	3 (8,3)
Liposarcoma	-	-	-	-	3	-	-	3 (8,3)
Carcinosarcoma	-	1	-	1	-	-	-	2 (5,6)
Neural sheath sarcoma	-	-	-	2	-	-	-	2 (5,6)
Osteosarcoma	-	-	-	-	-	2	-	2 (5,6)
Myxosarcoma	1	-	1	-	-	-	-	2 (5,6)
Ewing Sarcoma	-	-	-	1	-	-	-	1 (2,8)
Spindle cell Sarcoma	-	1	-	-	-	-	-	1 (2,8)
Non-classified Sarcoma	-	-	-	-	-	-	1	1 (2,8)
Total	8	5	7	5	8	2	1	36 (100)

On patient status in the last visit, we noticed that most of the cases (15, 41.6%) were live patients without disease evidence, followed by live patients with local disease (nine cases, 25%). The histological type most responsible for the higher number of deaths was the rhabdomyosarcomas, with four deaths (11.1%), while the dermatofibrosarcoma protuberans was the histological type with the highest number of live patients with no disease evidence, with five cases (13.8%). The face location had the highest number of deaths, with five cases (13.8%), and the tumor location with the highest number of patients without evidence of the disease was the scalp (five cases 13.8%). The sample distribution as to histological type, tumor location and patient status at the last visit can be seen on Table 3.

**Table 3:** Sample distribution according to histological type, location and status in the latest medical visit.

Patient status in the latest visit				
	Alive without the disease	Alive with local disease	Alive with metastasis	Death
Rhabdomyosarcomas				
Dermatofibrosarcoma	1	1	3	4
Fibrosarcoma	5	-	-	-
Malignant Fibro-histiocytoma	2	2	1	-
Liposarcoma	1	1	-	1
Carcinosarcoma	2	1	-	-
Neural sheath sarcoma	1	1	-	-
Osteosarcoma	-	2	-	-
Osteossarcoma	1	-	-	1
Ewing Sarcoma	-	-	-	1
Spindle-cell Sarcoma	-	1	-	-
Myxosarcoma	1	-	-	1
Non-classified Sarcoma	1	-	-	-
Location				
Face	2	1	-	5
Oral cavity	2	2	1	-
Scalp	5	1	1	-
Orbit	-	3	1	1
Neck	4	2	1	1
Gnathic bones	1	-	-	1
Parapharyngeal space	1	-	-	-

The most used treatment modes were surgery + radiotherapy + chemotherapy (tem cases, 27.8%) and surgery + radiotherapy (ten cases, 27.8%), as depicted on Table 4.

**Table 4:** Sample distribution according to treatment and patient status at the latest visit.

Patient status at the latest medical visit				
	Alive without the disease	Alive with local disease	Alive with metastasis	Death
Treatment				
Surgery	6	1	-	-
Radiotherapy	-	-	-	1
S + R	6	4	-	-
S+C	-	1	-	-
R+C	-	2	1	4
S+R+C	3	1	3	3
Total	15	9	4	8

S=Surgery, R= Radiotherapy, C= Chemotherapy. Source: research data

## DISCUSSION

Sarcomas represent a group of very rare diseases, with great diversity, which makes it difficult to run survey studies; thus restricting our knowledge on this type of disease.

Literature shows some survey studies involving head and neck sarcomas^1^, ^3^, ^4^, ^5^, ^6^, ^7^, ^8^, ^9^, ^11^, ^12^, ^14^, ^15^, ^16^, ^17^, ^18^, ^19^, ^20^, ^21^, ^22^ which investigate mainly patient profile and disease prognostic factors.

In this study, there was a higher prevalence in men, with a statistically significant difference, agreeing with other studies^9^, ^11^, ^16^, ^22^. Bentz et al.^19^ found very similar prevalence between the genders; and Bree et al.^1^ found a higher frequency among females. The men/women ratio found in this study was (1.76:1), similar to what has been reported by other authors, such as Le et al.^12^ and Singh et al.^22^, who found ratios of 1.9:1 and 2:1, respectively.

As far as age is concerned, there was a higher occurrence among patients between 20 and 59 years. The mean age of the patients was 39.7 (±25.10), similar to what was found by Dudhat et al.^16^, which was of 37 years, and the one reported by Gorsky and Epstein^11^, which was 40.4 years. In a thorough literature review, including studies published between 1972 and 2000, Mendenhall et al.^21^ found mean ages between 50 and 55 years. The literature shows that, in the head and neck sarcomas usually affect younger patients when compared to the squamous cell carcinomas^23^, including children and teenagers; and risk factors such as tobacco have not been associated with these tumors^3^.

The greatest involvement of the brown race in this study, representing 72.3% of the cases, can be explained by the large mix of ethnics found in northeastern Brazil. Arndt and Crist^24^ showed that the incidence of a specific type of sarcoma, the rhabdomyosarcomas, in white children is twice that in black children; nonetheless, most sarcoma studies do not show associations with skin color.

Noticing a balance between the number of patients from the capital city and those coming from the country side, we become concerned with the commuting of patients from the country side in order to have their treatment. In developing countries, like Brazil, specialized medical care is still short supply in the countryside, patients must travel to the capital in order to receive specialized care. We also stress that often times the patients must stay in support public housing, away from their homes and families, making their recovery even more difficult. Distance also impairs the long term follow up of these patients, whom sometimes waive their return visits. Besides the loss in treatment and proservation, all these difficulties hamper epidemiological studies, which demand long term follow up of these patients.

In the present study, we found 12 histopathological types of sarcomas, which show the large histological variability of these tumors. The World Health Organization defined more than 50 subtypes of sarcomas^2^. Lajer et al.^8^ found a number of patients similar to that of the present study (36); however, with 15 histopathology variations. The most common subtype in our study was the rhabdomyosarcomas, representing 25% of the cases, which was the same frequency found by Penel et al.^9^. Other studies showed lower prevalence: 8%^19^, 10%^20^, 16%^4^. Dermatofibrosarcoma protuberans and fibrosarcoma bear the second highest incidence, 13.8% of the cases each. Le et al.^12^ found prevalence of 6% and 15%, respectively for both subtypes.

As far as tumor location is concerned, the face and neck are the most affected regions, with 13.8% of the cases. Kraus et al.^18^ also found these two regions as the most frequent in the head and neck. Penel et al.^9^ found a greater involvement of the neck, with a frequency of 39.3%.

About patient status in the last visit, it was seen that 41.6% of the patients were alive and without evidence of the disease. This value is similar to the one found by Tran et al.^5^ in a survey involving 164 patients with head and neck sarcomas, in which the prevalence of live, disease-free patients was of 45.1%. The study from Nasri et al.^6^ found an incidence of 54%, and Huber et al.^14^ found 50%. In our study, live patients with local disease had a prevalence of 25%, similar to the findings from Tran^5^: 21.3%. Death happened to 22.2% of the cases, similar to the report from Huber^14^, who found 18.19%. Other older studies showed higher death rates, such as those from Tran^5^, Djkstra^15^ and Kraus^18^, who found prevalence of 33.5%; 34.5% and 33.3%, respectively, which lead us to believe that the progress in medicine has improved the prognosis of these patients.

Rhabdomyosarcomas was the histological type that had the highest number of deaths (11.1%). This subtype of sarcoma has a historically poor prognosis; nonetheless, progresses seen in therapy improved substantially the clinical outcome of these patients^7^. Dermatofibrosarcoma protuberans type had the highest number of live patients without evidence of disease (13.8% of the cases). According to Bree et al.^1^, this is usually a low grade neoplasm, and this classification is done according to the number of mitosis, nuclear pleomorphic, necrosis and cellularity. Low grade tumors have a better clinical and biological behavior, with better response to treatment and better prognoses. Sturgis et al.^7^ stated that the dermatofibrosarcoma protuberans has a locally aggressive behavior, but it usually does not metastasize.

The face represented the tumor location with the highest number of deaths (13.8%); and the highest number of live patients without the disease was those with scalp disease (13.8%). In the head and neck, a factor that really impairs sarcoma prognosis is location, since these tumors usually invade normal tissue, rendering proper resection difficult without causing significant functional and cosmetic deformities^12^. Thus, the tumor location impacts the selection of surgical options, the potential of obtaining free margins and the patient's functional condition^1^. This may explain the poor prognosis in facial sites; but the number of patients in this study and others already published is very low to consider location as a prognostic factor.

Since this is a rare disease, it is difficult to establish a standard treatment for head and neck sarcomas^4^. Treatment usually includes multiple treatment modes, especially when it is not possible to totally resect the tumor when it is near noble anatomical structures^10^; nonetheless, the best treatment option is total tumor resection^21^. In our study, most of the patients were treated with surgery + radiotherapy + chemotherapy, representing 27.8% of the cases, and surgery + radiotherapy, also with 27.8% of the cases. Other authors found lower frequencies; Penel et al.^9^ found 14.2% and 21.4% respectively, and Lajer et al.^8^, 16.6% and 13.8%, respectively. Most studies show a large frequency of patients who were submitted to surgery only, as the studies by Bentz et al.^19^, with 77% of patients, and that of Lajer et al.^8^, with 52.7%.

## CONCLUSIONS

The data in this study show that head and neck sarcomas are rare tumors with high histological variability, which may involve different anatomical sites. The most affected individuals were brown adult men, in the age range between 20 and 59, with mean age of 39.7. The predominant histological type was the rhabdomyosarcomas and the most common locations were the face and the neck. Most of the patients were live without evidence of disease in the last visit. The most common treatment modes were the combination of surgery + radiotherapy + chemotherapy and surgery + radiotherapy. Since these are rare and not well-known lesions, new epidemiological studies must be carried out, in order to provide us with more knowledge about the disease.

## References

[1] Bree R, Valk PVD, Kuik DJ, Diest PJV, Doornaert P, Buter J (2006). Prognostic factors in adult soft tissue sarcomas of the head and neck: A single-centre experience. Oral Oncol.

[2] Lahat G, Lazar A, Lev D. (2008). Sarcoma Epidemiology and Etiology: Potential Environmental and Genetic Factors. Surg Clin N Am.

[3] Gorsky M, Epstein JB. (1998). Head and neck and intra-oral soft tissue sarcomas. Oral Oncol.

[4] Yamaguchi S, Nagasawa H, Suzuki T, Fujii E, Iwaki H, Takagi M, Amagasa T. (2004). Sarcomas of the oral and maxillofacial region: a review of 32 cases in 25 years. Clin Oral Invest.

[5] Tran LM, Mark R, Meier R, Calcaterra TC, Parker RG. (1992). Sarcomas of the head and neck. Prognostic factors and treatment strategies. Cancer.

[6] Nasri S, Mark RJ, Sercarz JA, Tran LM, Sadeghi S. (1995). Pediatric Sarcomas of the Head and Neck Other Than Rhabdomyosarcoma. Am J Otolaryngol.

[7] Sturgis EM, Potter BO. (2003). Sarcoma of the head and neck. Curr Opinion Oncol.

[8] Lajer CB, Daugaard S, Hansen HS, Kirkegaard J, Holmgaard S, Christensen ME. (2005). Soft tissue sarcomas of the head and neck: a single-centre experience. Clin Otolaryngol.

[9] Penel N, Van Haverbeke C, Lartigau E, Vilain MO, Ton Van J, Mallet Y (2004). Head and neck soft tissue sarcomas of adult: prognostic value of surgery in multimodal therapeutic approach. Oral Oncol.

[10] Pellitteri PK, Ferlito A, Bradley PJ, Shaha AR, Rinaldo A. (2003). Management of sarcomas of the head and neck in adults. Oral Oncol.

[11] Gorsky M, Epstein JB. (2000). Craniofacial osseous and chondromatous sarcomas in British Columbia - a review of 34 cases. Oral Oncol.

[12] Le QT, Fu KK, Kroll S, Fitts L, Massullo V, Ferrell L (1997). Prognostic factors in adult soft tissue sarcoma of the head and neck. Int J Radiat Oncol Biol Phys.

[13] Hoffman HT, Robinson RA, Spiess JL, Buatti J. (2004). Update in management of head and neck sarcoma. Curr Opin Oncol.

[14] Huber GF, Mattews W, Dort JC. (2006). Soft-tissue sarcomas of the head and neck: a retrospective analysis of the Alberta Experience 1974 to 1999. Laryngoscope.

[15] Dijkstra MD, Balm AJM, Coevorden FV, Gregor RT, Hart AA, Hilgers FJ (1996). Survival of adult patients with head and neck soft tissue sarcomas. Clin Otolaryngol.

[16] Dudhat SB, Mistry RC, Varughese T, Fakih AR, Chinoy RF. (2000). Prognostic factors in head and neck soft tissue sarcomas. Cancer.

[17] Eeles RA, Fisher C, A'Hern RP, Robinson M, Rhys-Evans P, Henk JM (1993). Head and neck sarcomas: prognostic factors and implications. Br J Cancer.

[18] Kraus DH, Dubner S, Harrison LB (1994). Prognostic factors for recurrence and survival in head and neck soft tissue sarcomas. Cancer.

[19] Bentz BG, Singh B, Woodruff J, Brennan M, Shah JP, Kraus D. (2005). Head and neck soft tissue sarcoma: a multivariate analysis of outcome. Ann Surg Oncol.

[20] Chen AS, Morris CG, Andur RJ, Wernig JW, Villaret DB, Mendenhall WM. (2005). Adult head and neck soft tissue sarcomas. Am J Clin Oncol.

[21] Mendenhall WM, Mendenhall CM, Werning JW, Riggs CE, Mendenhall NP. (2005). Adult head and neck soft tissue sarcomas. Head Neck.

[22] Singh RP, Grimer RJ, Bhujel N, Carter SR, Tillman RM, Abudu A. (2008). Adult head and neck soft tissue sarcomas: treatment and outcome. Hindawi Publishing Corporation.

[23] Patel SG, Shaha AR, Shah JP. (2001). Soft tissue sarcomas of the head and neck: an update. Am J Otolaryngol.

[24] Arndt CAS, Crist WM. (1999). Commom musculoskeletal tumors of childhood and adolescence. N Engl J Med.

